# Supplementary material for: The Systems Biology Research Tool: evolvable open-source software
Source: BMC Syst Biol. 2008 Jun 29;2:55. doi: 10.1186/1752-0509-2-55 (PMC2446383; doi:10.1186/1752-0509-2-55)
Supplement: Additional file 1 — SBRT Archive. An archive of the current version of the Systems Biology Research Tool. [file 1752-0509-2-55-S1.zip › sbrt-1.4.0/doc/developers_guide/api/sbrt/kernel/math/package-tree.html]

sbrt.kernel.math Class Hierarchy


|  |  |  |
| --- | --- | --- |
| **Overview** \| **Package** \| Class \| Use \| **Tree** \| **Deprecated** \| **Index** \| **Help** | |  |
| **PREV**   **NEXT** | **FRAMES**    **NO FRAMES**     **All Classes** |  |


---


## Hierarchy For Package sbrt.kernel.math

**Package Hierarchies:**: All Packages

---

## Class Hierarchy

- java.lang.Object
  - sbrt.kernel.math.**AbstractLinearComb.Term**<V> (implements sbrt.kernel.math.LinearComb.Term<V>)
    - sbrt.kernel.math.**HashLinearComb.Term**<V>
    - sbrt.kernel.math.**AbstractPolynomial**<V> (implements sbrt.kernel.math.Polynomial<V>)
      - sbrt.kernel.math.**AbstractLinearComb**<V> (implements sbrt.kernel.math.LinearComb<V>)
        - sbrt.kernel.math.**HashLinearComb**<V>
      - sbrt.kernel.math.**DoubleEquiv** (implements sbrt.kernel.math.EquivTester<T>)- sbrt.kernel.math.**Interval** (implements java.io.Serializable)- sbrt.kernel.math.**IntervalComparer**- sbrt.kernel.math.**IntervalComparer.Diff**<V>- sbrt.kernel.math.**IntervalEquiv** (implements sbrt.kernel.math.EquivTester<T>)- java.lang.Throwable (implements java.io.Serializable)
                  - java.lang.Exception
                    - java.lang.RuntimeException
                      - sbrt.kernel.math.**ConstraintViolationException**- sbrt.kernel.math.**ExceededLimitException**- sbrt.kernel.math.**IllegalBoundsException**- sbrt.kernel.math.**InfeasibleSolutionException**- sbrt.kernel.math.**InvalidPointException**- sbrt.kernel.math.**MissingVariableException**- sbrt.kernel.math.**UnboundedVariableException**
                  - sbrt.kernel.math.**VectorComparer**- sbrt.kernel.math.**VectorComparer.Diff**<V>

## Interface Hierarchy

- sbrt.kernel.math.**EquivTester**<T>- java.lang.Iterable<T>
    - sbrt.kernel.math.**LinearComb**<V> (also extends sbrt.kernel.math.Polynomial<V>)- sbrt.kernel.math.**Polynomial.Term**<V>
        - sbrt.kernel.math.**LinearComb.Term**<V>- sbrt.kernel.math.**QuadraticExpr.Term**<V>
    - sbrt.kernel.math.**MathExpr**<V>
      - sbrt.kernel.math.**Polynomial**<V>
        - sbrt.kernel.math.**LinearComb**<V> (also extends java.lang.Iterable<T>)- sbrt.kernel.math.**QuadraticExpr**<V>

---


|  |  |  |
| --- | --- | --- |
| **Overview** \| **Package** \| Class \| Use \| **Tree** \| **Deprecated** \| **Index** \| **Help** | |  |
| **PREV**   **NEXT** | **FRAMES**    **NO FRAMES**     **All Classes** |  |


---
